# Stimulatory Effects of Acibenzolar-*S*-Methyl on Chlorogenic Acids Biosynthesis in *Centella asiatica* Cells

**DOI:** 10.3389/fpls.2016.01469

**Published:** 2016-09-28

**Authors:** Efficient N. Ncube, Paul A. Steenkamp, Ntakadzeni E. Madala, Ian A. Dubery

**Affiliations:** ^1^Department of Biochemistry, University of JohannesburgAuckland Park, South Africa; ^2^Council for Scientific and Industrial Research Biosciences, Natural Products and Agroprocessing GroupPretoria, South Africa

**Keywords:** acibenzolar-*S*-methyl (ASM), *Centella asiatica*, chlorogenic acids, elicitation, quinic acid, shikimic acid

## Abstract

*Centella asiatica* is a perrenial herb that grows in tropical regions with numerous medicinal properties mostly attributed to the presence of pentacyclic triterpenoids. Interestingly, this plant also possess a significant amount of phenylpropanoid-derived chlorogenic acids (CGAs) that have recently been reported to confer neuroprotective properties. In a biotechnological attempt to increase the biosynthesis of CGA-derivatives in cultured *Centella* cells, acibenzolar-*S*-methyl was applied as a xenobiotic inducer in combination with quinic acid and shikimic acid as precursor molecules. Applying a semi-targeted metabolomics-based approach, time and concentration studies were undertaken to evaluate the effect of the manipulation on cellular metabolism leading to CGA production. Phytochemical extracts were prepared using methanol and analyzed using a UHPLC-qTOF-MS platform. Data was processed and analyzed using multivariate data models. A total of four CGA-derivatives, annotated as *trans*-5-feruloylquinic acid, 3,5 di-caffeoylquinic acid, 3,5-*O*-dicaffeoyl-4-*O*-malonylquinic acid (irbic acid) and 3-caffeoyl, 5-feruloylquinic acid, were found to be upregulated by the acibenzolar-*S*-methyl treatment. To the best of our knowledge, this is the first report on the induction of CGA derivatives in this species. Contrary to expectations, the effects of precursor molecules on the levels of the CGAs were insignificant. However, a total of 16 metabolites, including CGA derivatives, were up-regulated by precursor treatment. Therefore, this study shows potential to biotechnologically manipulate *C. asiatica* cells to increase the production of these health beneficial CGAs.

## Introduction

In order to enhance secondary metabolite production, the biotechnological manipulation of cultured cells with plant signaling molecules and elicitors is becoming widespread in plant research (Bourgaud et al., 2001; Hellwig et al., 2004). These systems allow for a number of plant bioactive constituents to be rapidly biosynthesized, extracted and analyzed. Exogenous application of such elicitors has been shown to induce metabolic changes by upregulating or downregulating certain defense-associated pathways in plants (Hayat et al., 2010). An added advantage of plant cell culture systems is that they are economical to grow and maintain (Rao and Ravishankar, 2002; Hellwig et al., 2004).

Acibenzolar-*S*-methyl (ASM) or *S*-methyl 1,2,3-benzothiadiazole-7-carbothioate is a synthetic agrochemical (Actigard, Bion and Bendicar) and a functional analog of the phytohormone salicylic acid (SA) (Hammerschmidt, 2009; Vos et al., 2013). Similarly to SA, ASM is a highly potent activator of SAR that upregulates the expression of pathogenesis-related (PR) genes and ultimately results in the production of defense-related compounds associated with the phenylpropanoid pathway (Herman et al., 2007; Hammerschmidt, 2009; Conrath, 2011; Hong et al., 2011). In that regard, previous reports have suggested that ASM either activates the systemic acquired resistance (SAR) pathway downstream or directly at the SA site of action (Oostendorp et al., 2001). Interestingly though, ASM appears to induce a stronger resistance against infection than SA (Du et al., 2012; Sillero et al., 2012). Previous reports have shown that ASM induces resistance against a wide range of pathosystems in both monocots and dicots (Oostendorp et al., 2001; Hong et al., 2011; Walters et al., 2013). Our previous work has reported on the upregulation of chlorogenic acid (CGA) derivatives upon elicitation of tobacco cells with both SA and ASM (Mhlongo et al., 2014; Mhlongo et al., 2016). However, an attempt to increase the biosynthesis of CGAs in *Centella asiatica* with the exogenous application of SA proved ineffective (Ncube et al., 2016).

Quinic- and shikimic acids (QA, ShA) are alicyclic acids that exist in equilibrium and are primary intermediates of the shikimate pathway leading to the phenylpropanoid pathway (Johansson et al., 2005; Ali et al., 2007; Ghosh et al., 2012). Therefore, the addition of these precursor alicyclic acids to existing metabolic networks in plant cells (Stockigt and Zenk, 1974; Vogt, 2010; Ghosh et al., 2012) could increase the biosynthesis of CGAs. Such a precursor feeding approach, is an attempt to increase the yield of the final secondary metabolite and has been effectively carried out by exogenously supplying pathway intermediates to plant cell cultures (Rao and Ravishankar, 2002; Verpoorte et al., 2002; Jackson and Attalla, 2010; Murthy et al., 2014).

*Centella asiatica*, a member of the Umbelliferae order and Apiaceae family (Shukri et al., 2011; Gallego et al., 2014), is a perennial herb that grows in tropical, pan-tropical and/or sub-tropical regions throughout the world (James and Dubery, 2009). The herb has been well documented mostly due to its medicinal properties but also as a nutritional and industrial crop (Hashim, 2011; Gallego et al., 2014). Various therapeutic properties of the herb have been extensively documented (James and Dubery, 2009; Gallego et al., 2014; Gupta et al., 2014), where recently, it has also been reported to possess neuroprotective properties in humans and animal models (Gao et al., 2009; Omar et al., 2011; Kim et al., 2013; Gallego et al., 2014; Prasad et al., 2014). The various bio-active properties of *C. asiatica* are attributed to the composition of its secondary metabolite profile. In this regard, it contains a great percentage of pentacyclic triterpenoids (asiatic acid, madecassic acid, asiaticoside, madecassodise) collectively known as centelloids (James and Dubery, 2009; James et al., 2013). In addition to the centelloids, other terpenoid metabolites with bio-activities are also found (Oyedeji and Afolayan, 2005). Analysis of its essential oils in previous studies has revealed the presence of sesquiterpenoids (Brown et al., 2011; Oyedeji and Afolayan, 2005). In addition to the well-described terpenoids, *C. asiatica* also accumulates phenolic compounds with therapeutic properties such as CGAs (Gray et al., 2014) that have been less well studied.

Recent reports have identified CGAs from *C. asiatica* as beneficial in the treatment of, or prevention of age-related degenerative diseases, including Alzheimer’s disease (Gao et al., 2009; Omar et al., 2011; Gallego et al., 2014; Prasad et al., 2014). CGAs are important constituents of plant secondary metabolites and consists of hydroxycinnamic acids (HCAs, e.g., caffeic acid) esterified to quinic acid. The abundant cinnamates found in nature include ρ-coumaric-, caffeic- and ferulic acids that result in the formation of the most common CGAs: ρ-coumaroylquinic-acid (ρ*-*CoQA), caffeoylquinic acid (CQA) and feruloylquinic acid (FQA) respectively (Clifford, 1999; Marques and Farah, 2009).

In this communication we report on the CGA and HCA profiles of *C. asiatica* cells and the ability of ASM and precursor molecules (QA and ShA) to enhance the levels of CGAs.

## Materials and Methods

### Cell Culture Preparation, Viability Assay and Elicitation

Cell cultures of *C. asiatica* were established as previously reported (James and Dubery, 2011; James et al., 2013). Treatment of the cells was carried out in a sterile laminar flow hood. *C. asiatica* cells, grown in Murashige and Skoog (MS) medium with MS vitamins and containing 1 μM 2,4-dichlorophenoxyacetic acid and 0.5 μM benzyl aminopurine, 1 g/L casein hydrolysate and 30 g/L sucrose (pH 5.8) (James et al., 2013), were used 3 days after sub-culture.

To investigate any possible detrimental effects of ASM on the cells, viability was determined using the Alamar Blue assay (Byth et al., 2001). For this assay, the required amount of a 100 X stock solution of ASM (Syngenta, Basel, Switzerland) for concentrations ranging from 0 to 1000 μM was added to the cells for an incubation periods of 12 and 24 h.

Before treatment, cells from different flasks were combined to ensure a homogenous culture. The combined suspension of cells was divided into 24 × 20 mL aliquots (12 for the concentration study and 12 for the time study) into sterile 50 mL Erlen-Meyer flasks. For elicitation, ASM was added (from a 80 mM stock solution) to reach final concentrations of 100, 200, and 300 μM, (i.e., 25, 50, and 75 μL respectively) for the ASM concentration study. The flasks were then capped and placed on an orbital shaker at 130 rpm in a plant tissue culture room at 23°C for 24 h (Ncube et al., 2014). For the concentration study, the cells were harvested after 24 h incubation time. For the time study, cells were harvested after 0-, 6-, 12-, and 24 h time points.

Cells were harvested using a vacuum filtration system on 55 mm filter paper (Millipore, Billerica, MA, USA) to remove the original culture medium. The cells were weighed, transferred to sterile Falcon tubes and washed with 40 mL cold, sterile, MS basal salts medium (without any vitamins, hormones and inducing agents) and filtered again as described.

Once optimal conditions were determined, a precursor feeding study was carried out where the cells were treated with ASM, QA (Alfa Aesar, Heysham, England), ShA (Alfa Aesar, Heysham, England) and combinations of ASM and QA and ShA. Thus, a concentration of 300 μM ASM and 100 μM QA and 100 μM ShA was used to treat the cells as described above. QA and ShA were added 6 h after the initial addition of ASM. At all instances, the acidic precursor molecules were neutralized using Tris(hydroxymethyl)aminomethane to pH 6.5 and sterilized through 0.2 μm nylon filters.

The experimental design consisted of at least three biological replicates for each experimental combination in order to generate the required number of data points for downstream metabolomic analyses.

### Metabolite Extraction and Concentration

Two g of the freshly collected and washed cells was weighed out and re-suspended in methanol at a ratio of 1:10 (g of cells: ml of methanol) in a 50 mL Falcon tube. A probe sonicator (Bandelin Sonopuls, Berlin, Germany) was used to homogenize the cells at 55% power for 15 s with four cycles. The homogenates were then centrifuged in a benchtop centrifuge at 5100 × *g* for 15 min at room temperature. The methanol was evaporated by rotary evaporation at 50°C to approximately 1 mL. The residues were taken from the round bottom flasks and transferred into 2 mL Eppendorf tubes and dried to completeness at 55°C overnight. The remaining dry residues of all the samples were reconstituted in 500 μL of 50% aqueous methanol and placed in pre-labeled sample glass vials fitted with 300 μL inserts and unslitted caps.

### UHPLC-qTOF-MS Analyses

Ultra-high performance liquid chromatography mass spectrometry (UHPLC-MS) analyses were carried out on an Acquity UHPLC system connected to a photodiode array (PDA) detector as well as a SYNAPT G1 high definition (HD) MS quadrupole time-of flight (QTOF) mass spectrometer (Waters Corporation, Milford, MA, USA). A Waters Acquity UHPLC column (CSH-C18, 150 mm × 2.1 mm, 1.7 μm) thermostatted at 60°C, was used to chromatographically separate the extracts. A binary solvent gradient was utilized, comprised of water with 0.1% formic acid (Romil, Cambridge, UK) (eluent A) and acetonitrile (Romil, Cambridge, UK) with 0.1% formic acid (eluent B). The flow rate was set at 0.4 mL min^-1^ whilst the injection volume was set and 3 μL. Initial conditions were 5% B, the conditions were then changed from 5 to 90% B over 0.1 – 16 min and held constant at 90% B for 1 min over 16 – 17 min. The gradient was dropped to the initial conditions for 1 min over 17 – 18 min and the initial conditions held for 2 min, resulting in a run time of 20 min. A PDA detector set at 200–500 nm (1.2 nm resolution) with a sampling rate of 20 points/s, was used to monitor the chromatographic elution.

Post-PDA detection, the SYNAPT G1 HD-MS (Waters Corporation, Manchester, UK) operated in electrospray ionisation (ESI) positive and negative modes, was used to detect the analytes of interest.

Based on literature, previous metabolite fingerprinting studies of phenolic compounds and -derivatives have mostly been carried out in ESI negative mode (Clifford et al., 2003; Jaiswal et al., 2014). As the focus of this current work is to report the effect of SA on the phenylpropanoid pathway, particularly chlorogenic acid profiles, in *C. asiatica* cells, only data obtained from ESI negative ionization mode was processed for further analyses. The MS settings were as follows: source temperature of 120°C, capillary voltage of 3 kV, sample cone voltage of 60 V, extraction cone voltage of 5 V, collision energy of 3 V, detector voltage of 1650 V, scan time of 0.2 s, interscan time of 0.02 s, *m/z* range of 100–1100, in centroid data mode. The desolvation gas used was high purity nitrogen at 450°C and cone gas at 50 Lh^-1^. Leucine enkephalin (556.2771 Da) was used as a calibrant (566.3 ng μL^-1^) at a flow rate of 0.1 mL min^-1^ and a mass window of 5 mDa to achieve high mass accuracy. For data acquisition pooled samples (QC) were used for quality control checks. Sample acquisition was randomized and the QC sample analysed every 10 injections to monitor and correct changes in the instrument response.

To assist with the downstream annotation and identification of the biomarkers associated with these treatments, the MS experiment file was setup to perform unfragmented as well as five fragmenting experiments (MSE) simultaneously. Ion fragmen tation was performed by increasing the in-source collision energy (3–30 eV) (Madala et al., 2014b; Ncube et al., 2014).

### Multivariate Data Analysis

#### PCA and OPLS-DA Modeling

The MarkerLynx^TM^ application manager of the MassLynx^TM^ software (Waters Corporation, Manchester, UK) was used for raw UHPLC-MS pre-processing (matrix creation) with the software parameters set as: 3–13 min retention time (Rt) range of the chromatogram, mass range 100–900 Da, mass tolerance 0.05 Da, mass window 0.05 Da and a Rt window of 0.20 min. The data matrices obtained were exported to SIMCA (Soft Independent Modelling of Class Analogy) software, version 13.0.2 (Umetrics, Umea, Sweden) for multivariate data analysis to generate models such as principal component analyses (PCA) derived score plots and hierarchical clustering analysis (HCA). Orthogonal projection to latent structures-discriminant analysis (OPLS-DA) derived score plots and S-plots were used to identify signatory biomarkers perturbed by or associated with the treatments (Tugizimana et al., 2012). *Pareto-* scaled data was used to generate all models. The quality of PCA models was determined by (i) cumulative modelled variation in the X matrix, R^2^X (cum) also known as the goodness-of-fit parameter and (ii) the predictive ability parameter Q^2^ (cum). Values close to 1 indicate a robust model (Supplementary Table S1). The OPLS-DA models were statistically reliable with CV-ANOVA *p*-value of ≤0.001 (Supplementary Table S2).

#### XCMS Cloud Plot Analysis

The LC-MS data were further analyzed also using XCMS (various forms of chromatography-mass spectrometry) software package^1^ based on the R statistical language (Benton et al., 2008; Gowda et al., 2014). The parameters for XCMS parameters were as follows: (i) Feature detection was performed with *m/z* deviation of 15 ppm, maximum peak width of 20 s, (ii) Rt correction was achieved using Orbiwarp method, (iii) Alignment was performed using minimum fraction of samples of 0.5, Rt deviation of 5 s, *m/z* window of 0.015, (iv) statistical test was performed using the unpaired parametric *t*-test, at a *p*-value threshold of 0.05 and a fold change threshold of 1.5, (v) annotation was performed with an *m/z* absolute error of 0.002.

### Relative Concentration Determination

The SPSS software package (IBM^2^) was used to generate the box and whisker plots for the relative concentration of the annotated metabolites based on the area under the peak.

### Metabolite Annotation

Metabolite annotation was at the level 2 of the Metabolomic Standards Initiative (MSI) (Sumner et al., 2007). The mass fragmentation patterns of the annotated/tentatively identified metabolites were obtained by generating spectra using alternating collision energies (CE). Furthermore, these annotations were confirmed by generating further spectra using an optimized in-source collision induced dissociation (ISCID) approach for the annotation of CGAs (Ncube et al., 2014). Stable fragmentation patterns were experimentally optimized by changing the trap collision energy (3–60 eV) and the cone voltage (10–100 V) until the formation of the following stable ions: Q1[quinic acid-H]^-^ at *m/z* 191, C1[caffeic acid-H]^-^ at *m/z* 179, Q2[quinic acid-H-H_2_O]^-^ at *m/z* 173 and C2 [caffeic acid-H-CO_2_]^-^ at *m/z* 135. Thus, the obtained fragmentation patterns were correlated to proposed structures and were confirmed by comparison to literature. In order to confirm the annotation of the signatory biomarkers, the available authentic standards included *trans*-caffeoylquinic acids (3-, 4-, and 5-CQA) and dicaffeoylquinic acids (3,4-diCQA, 3,5-diCQA, 1,3-diCQA, 1,5-diCQA, and 4,5-diCQA) obtained from Phytolab (Vestenbergsgreuth, Germany) were also used. Moreover, the molecular formulae (MF) of the pseudo-molecular ions ([M-H]^-^) representing any possible metabolite were computed and searched on online databases such as Chemspider^3^, Dictionary of Natural Products^4^, knapsack^5^ as well as the Taverna work-bench^6^. The latter is based on the PUTMEDID_LCMS metabolite identification workflows that entail correlation analysis, metabolic feature annotation and metabolite annotation (Brown et al., 2011; James et al., 2013).

## Results and Discussion

In this study, a semi-targeted (with the focus on mid-polar compounds, particularly phenylpropanoids and CGAs) LC-MS based metabolomic approach was employed to determine the metabolic response of *C. asiatica* cells to ASM as an inducer, in combination with precursors QA and ShA as well as to the latter individual precursors without a pre-induction step. Initially, viability assays were performed to ensure that the concentrations used were not detrimental to cell viability and to determine thresholds of possible toxicity. The optimal conditions (>90% cell viability) with regards to incubation time period of ASM-treatment (12 and 24 h) and concentration of treatment (0 – 300 μM) were established based on the Alamar Blue assay (data not shown) (Byth et al., 2001; James et al., 2013).

**FIGURE 1:**
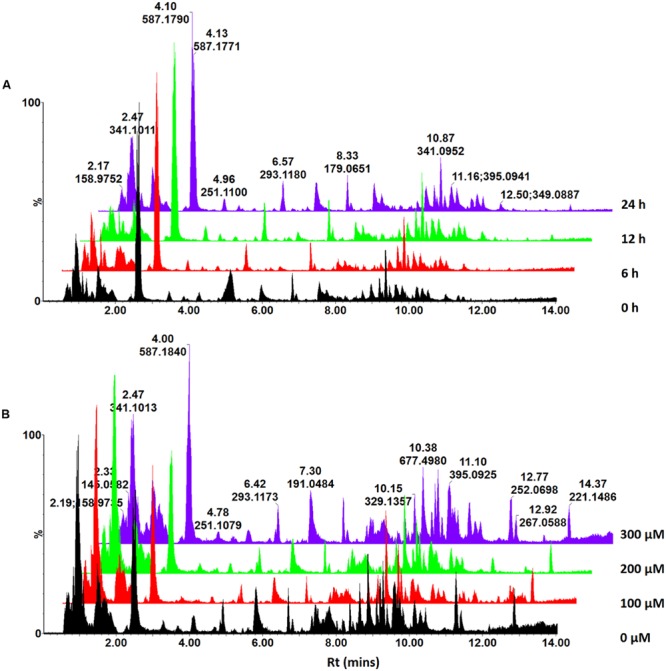
**UHPLC-MS analysis of ASM-treated *C. asiatica* cells.** Base peak intensity (BPI) chromatograms obtained from the time – **(A)** and concentration study **(B)**. A concentration of 300 μM ASM was used in the time study whereas the cells were incubated for 24 h for the concentration study. The *Y*-axis represents relative abundance and the chromatograms were offset along the *X*-axis with 0.5 min intervals for comparison.

Thus, time- and concentration studies were carried out as shown in the chromatographic analyses in **Figures 1A,B** respectively. As mentioned, only the ESI-negative mode data was further processed due to the better ionization of the phenolic acids in negative mode.

### Chromatographic Analyses

In our previous communication (Ncube et al., 2016), it was reported that *C. asiatica* cells were not responsive to treatment with SA in order to increase the biosynthesis of CGA derivatives. However, since ASM is reported to induce a stronger response than SA (Herman et al., 2007; Hammerschmidt, 2009; Conrath, 2011; Hong et al., 2011), the treatment with this inducer was expected to result in increased biosynthesis of these metabolites. The MS chromatograms indicate time- (**Figure 1A**) and concentration dependent (**Figure 1B**) metabolic responses to ASM treatment of *C. asiatica* cells. The concentration- and time studies were conducted to investigate the optimal conditions for treatment of the cells. However, as these differences were not pronounced and the elicitation did not result in the biosynthesis of new CGA derivatives, it was attempted to further increase the biosynthesis of CGA metabolites using a precursor feeding approach. Thus, treating the cells with the combination of the inducer and the precursors should have ideally enhanced the production of CGA derivatives, as a result of increased pools of readily available QA and caffeic acid derivatives (Johansson et al., 2005; Ali et al., 2007; Ghosh et al., 2012).

**FIGURE 2:**
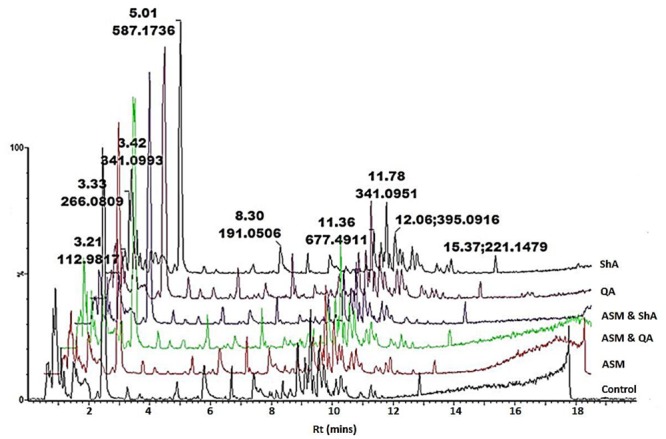
**UHPLC-MS analysis of precursor feeding of *C. asiatica* cells.** BPI chromatograms obtained from the control (non-treated) cells, cells treated with ASM, ASM and QA, ASM and ShA, QA as well as treated with ShA are shown. The concentration of ASM and the precursors was 300 and 100 μM respectively, whereas at all instances the cells were incubated for 24 h post treatment. The *Y*-axis represents relative abundance and the *X*-axis of the chromatograms were offset with 0.5 min.

Similarly, the presence/absence of some peaks (**Figure 2**) was indicative of differential effects on the metabolism of *C. asiatica* in response to the various treatments. A closer inspection of the differences/similarities between the different treated samples was carried out by comparing the non-treated with three conditions (e.g., control vs. ASM, [ASM + QA], [ASM + ShA], **Supplementary Figure S1**). However, due to the complexity of LC-MS chromatograms further analyses were carried out by processing the data using diverse chemometric-based multivariate data analysis tools.

### Multivariate Data Analysis

#### PCA and OPLS-DA Modeling

Principal component analyses is a non-supervised model that reduces the multi-dimensionality of complex data from analytical platforms. Thus, it depicts a global overview of all the similarity and/or dissimilarities between different treated sample groups (Trygg et al., 2007; Tugizimana et al., 2013; Eriksson et al., 2014). As such, the models facilitated descriptive visual evaluation of the effect of each treatment condition on *C. asiatica* cells. On the other hand, OPLS-DA is a supervised model that allowed the extraction of the significant biomarkers responsible for the separation of the different treated sample groups (Yamamoto et al., 2009; Tugizimana et al., 2013; Saccenti et al., 2014).

**FIGURE 3:**
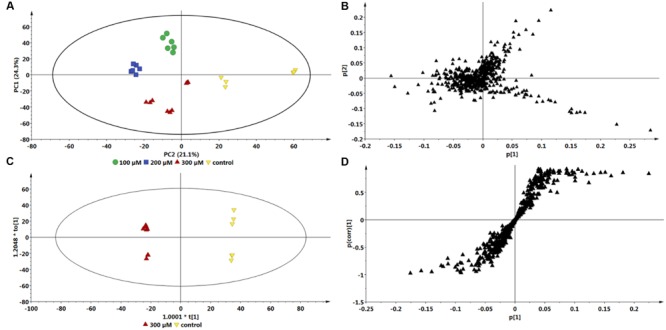
**Multivariate data models of changes occurring in *C. asiatica* cells over time in response to ASM as inducer.** Shown are the PCA scores- **(A)** and loadings plot **(B)**, OPLS-DA scores- **(C)** and S-plot **(D)**. Compared to the 0 h control extracts, there is a different sample clustering on the PCA scores plot for the 24 h extracts, with the 6 h and 12 h extracts not well separated. The QC (pooled) samples all grouped close to the center in the scatter plot (not shown). The ions on the right quadrant of the OPLS-DA S-plot were extracted as the significant biomarkers reflecting the metabolic effect of ASM on the *C. asiatica* cells. The S-plot is based on the optimal incubation period (24 h). The models were *Pareto* scaled.

Based on the chromatograms (**Figure 1**), the PCA score plot (**Figure 3A**) reveals that there is a time-dependent response of *C. asiatica* cells to ASM-treatment as reflected in the clustering at different coordinates of samples harvested at different time points, whereas the loadings plot shows each variable (*m/z* and Rt) representing each ion (**Figure 3B**). The metabolome profile of the cells is already changed at 6 h post-treatment. In addition, the close clustering of the sample groups of the cells harvested 6- and 12 h post-treatment implies ongoing changes, indicative of an early response which progresses to result in a distinctive cluster at 24 h post treatment (OPLS-DA scores-plot, **Figure 3C** and S-plot, **Figure 3D**).

**FIGURE 4:**
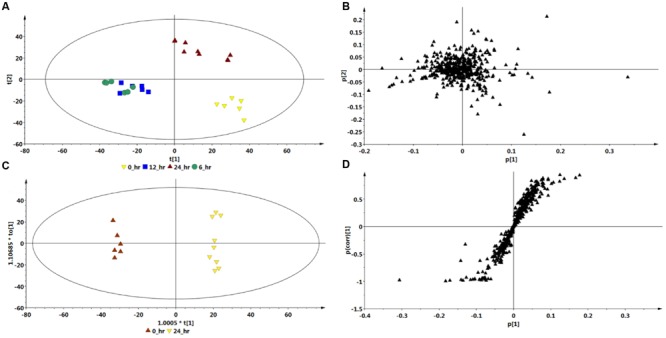
**Multivariate data models of changes occurring in *C. asiatica* cells in response to increasing concentrations of ASM.** Shown are the PCA scores- **(A)** and loadings plot **(B)**, OPLS-DA scores- **(C)** and S-plot **(D)**. There is different (concentration-dependent) sample clustering on the PCA scores plot. The S-plot is based on the optimal concentration (300 μM ASM). The models were *Pareto* scaled.

Multivariate data analyses of changes occurring in *C. asiatica* cells in response to increasing concentrations of ASM are shown in **Figure 4**. The PCA scores plot (**Figure 4A**) reflect concentration-dependent metabolic response of *C. asiatica* cells to ASM-treatment as seen on the chromatograms (**Figure 2**), whereas the loadings plot shows each variable (*m/z* and Rt) representing each ion (**Figure 4B**).

Based on the same data sets, the OPLS-DA model of control vs. 300 μM ASM treatment (**Figure 4C**) allows for the identification of the features (*m/z* ions at specific Rts) or biomarkers associated with the response of the cells towards ASM (**Figure 4D**).

Comparison of the S-plots generated to illustrate the separation at other time points (0 vs. 6 h and 0 vs. 12 h, data not shown) and concentration points (0 vs. 100 μM and 0 vs. 200 μM, data not shown) projected a similar pattern; i.e., showed the same significant biomarkers. However, as mentioned, the conditions that resulted in a greater response was at the highest concentration (300 μM) and the longest incubation period (24 h). The ions that are positively correlated to ASM-treatment on the OPLS-DA derived S-plot (**Figure 3D** and **4D**) are similar to those reported in the SA-treated *C. asiatica* cells (Ncube et al., 2016), and the same set of ions are defined as signatory biomarkers for both time- and concentration studies. However, contrary to the results obtained from the SA study (Ncube et al., 2016), the ASM-treatment resulted in the up-regulation/ increased biosynthesis of the CGA- derivatives represented by those ions.

#### XCMS Analysis

**FIGURE 5:**
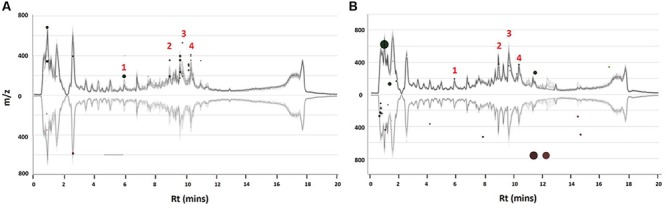
**XCMS interactive Cloud plot.** Shown are plots of the time- (0 vs. 24 h) **(A)** and concentration study (0 vs. 300 μM) **(B)** of ASM-treated *C. asiatica* cells. The Cloud plots are based on the optimal incubation period (24 h) and concentration of 300 μM respectively. Each dot represents a feature i.e., an ion with its *m/z* and Rt. The features in the upper section represent the up-regulated biomarkers whereas the down-regulated biomarkers are shown in the bottom section. The annotated biomarkers 1–4 were statistically significant at *p* ≤ 0.05.

The UHPLC-MS generated data was further analyzed using XCMS online to compliment the OPLS-DA derived S-plots (**Figures 3D and 4D**) as it also allows for the extraction of statistical significant biomarkers. Similarly, the four (1–4) upregulated biomarkers with *m/z* = 367.0994, 515.1201, 601.1159, and 529.1414 of Rts = 7.56, 8.99, 9.56, and 10.46 min respectively, as per the S-plot, were seen on the Cloud plots as well (**Figure 5**). It is also interesting to note that these biomarkers appear to be responsive to the time - and the concentration of the treatment. These results also confirm that incubating the cells with 300 μM ASM for 24 h results in a change in the metabolite profile of *C. asiatica* accompanied by enhancement of CGA derivatives. Thus, these metabolites (**Table 1**) were tentatively annotated and relatively quantified in Sections “Metabolite Annotation” and “Relative Concentration of CGA Biomarkers,” respectively.

**Table 1:** Tandem MS-based annotation of four chlorogenic acids biomarkers associated with treatment of *C. asiatica* cells with 300 μM ASM for 24 h.

	Rt (min)	[M-H]^-^(*m/z*)	Product ions (*m/z*)	Calculated mass	Putative identification
1	7.56	367.0994	191.0510, 173.0417 134. 0376	368.3353 8.5 ppm^∗^	*trans*-5-Feruloyl-quinic acid (C_17_H_20_O_9_)
2	8.99	515.1201	353.0836, 191.0497, 179.0289, 135.0408	516.4509 7.9 ppm^∗^	3,5 di-Caffeoylquinic acid (C_25_H_24_O_12_)
3	9.56	601.1159	395.0889, 353.0790, 233.0566, 191.0480, 179.0318, 135.0349	602.5370 7.8 ppm^∗^	3,5-O-di-Caffeoyl-4-O-malonyl quinic acid (Irbic acid) (C_28_H_26_O_15_)
4	10.46	529.1414	367.1035, 353.0860, 191.0526, 179.0309, 135.0395	530.4927 6.3 ppm^∗^	3-Caffeoyl, 5-feruloylquinic acid (C_26_H_26_O_12_)

*^∗^Difference between calculated and experimentally found masses, expressed as parts per million.*

#### Precursor Feeding Studies

**FIGURE 6:**
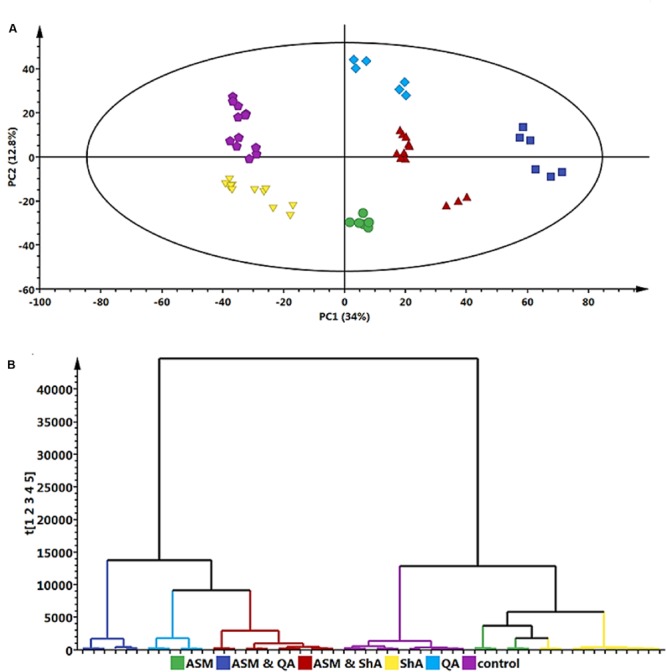
**Principal component analyses – derived models of the precursor (QA and ShA) feeding study of *C. asiatica* cells.** A concentration of 300 μM ASM and 100 μM QA and 100 μM ShA was used to treat the cells as described. QA and ShA were added 6 h after the initial addition of ASM. Shown are Scores- **(A)** and HCA plots **(B)** with the same color coding to indicate the different treatments. The QC (pooled) samples all grouped close to the center in the scatter plot (not shown). The sample grouping on the score plot clearly indicated differential effects of each treatment to the cells. The HCA complimented what could be observed on the score plot by providing the inter-relationships between the different treated samples and also indicated the samples could be separated in two major groups.

Hierarchical cluster analysis (HCA) provides a clearer overview of the relationship between groups (Madala et al., 2014a). **Figures 6A,B** indicates that extracts from the ASM treated cells and the extracts from cells treated with ShA alone were similar, and clustered close to the control (non-treated) cells. Overall, the treatment of the cells with ASM relatively has the least effect on the metabolite profile of the cells as these samples appear closely related to the control (non-treated cells). However, the combined treatments of [ASM + QA] and [ASM + ShA] exhibited clear differences compared to the control cells, indicating that the precursor feeding was significant in contributing to the differential clustering.

The differences between the QA and ShA treatments are noteworthy: the addition of ShA as a precursor seemingly resulted in less pronounced effects on the metabolic response of the cells. On the other hand, QA seems to result in a more pronounced effect on the metabolite profile of the cells as both the precursor molecule and the combination with ASM [ASM + QA], cluster the furthest from the control samples. Moreover, the [ASM + QA] combination appears to result in a similar response to just the QA alone, but the [ASM + ShA] combination treatment is significantly different from the individual ASM and ShA treatments of the cells (**Figure 6B**).

Accordingly, these findings show that QA and ShA have differential effects on *C. asiatica* cells, although they are both primary intermediates contributing to the phenylpropanoid pathways. As such, a study by Sonnante et al. (2010) reported that hydroxycinnamoyl-CoA quinate hydroxycinnamoyl transferase (HQT) isolated from Artichoke shows higher affinity for QA over ShA. However, these enzymatic dynamics have not been studied for *C. asiatica*. A separate study elsewhere confirmed that although QA and ShA are interconvertible, albeit the reation equilibration is highly dependent on a number of contributing environmental factors (Knop et al., 2001). Therefore, the clustering observed in (**Figure 6B**) indicates that the underlying metabolic pathways are complex and further studies are needed in order to unravel them.

In order to extract the significant biomarkers associated with each of these groups, OPLS-DA models comparing non-treated and each precursor treatment were generated (**Supplementary Figure S2**).

### Metabolite Annotation

#### ASM Treated Cells

The annotation of the signatory biomarkers (1–4) extracted from the OPLS-DA derived S-plot (**Figures 3D** and **4D**) and XCMS cloud plots (**Figure 5**) was validated in a similar manner as previously described (Ncube et al., 2016). Therefore, the tentative annotation (**Table 1**) was carried out following MSI level 2 (i.e., putatively identified, comparison of physicochemical properties and/or spectral similarity with public or commercial spectral libraries without authentic chemical standard) (Sumner et al., 2007) using an optimized UHPLC-QTOF-MS ISCID approach (Ncube et al., 2014) that has proved to effectively overcome the analytical challenges posed by the geometric and positional isomerism of these compounds (Jaiswal et al., 2014). The annotation followed the hierarchical scheme for the identification of CGAs by (Clifford et al., 2003). Here, the generation of fragmentation patterns using tandem MS (MS^2^), facilitated the monitoring of molecules containing a cinnamic acid moiety. For instance, metabolites with a quinic acid moiety were characterized by their fragmentation patterns with ion peaks representing Q1 [quinic acid-H] at *m/z* 191 and Q2 [quinic acid-H_2_O] at *m/z* 173 wherein they were also considered for CGA derivatives along with other ion peaks representing C1 [caffeic acid-H] at *m/z* 179 and C2 [caffeic acid-CO_2_] at *m/z* 135 respectively. Where available, authentic standards (particularly 3- and 5-CQA, 3,5- and 3,4-di-CQA for the current study) were also used to confirm the annotation of the signatory biomarkers. Thus, a total of four biomarkers (from the S-plots and XCMS cloud plots, **Figures 3D**, **4D**, and **5**, respectively) were annotated as described below.

As mentioned, the annotated CGA derivatives (**Table 1**) were also reported as significant biomarkers in the SA study (Ncube et al., 2016), and their detailed characterization is supplied in the **Supplementary Figure S3**.

#### Precursor (QA and ShA) Treated Cells

Similarly, the annotation of CGAs was performed following the hierarchical classification scheme (Clifford et al., 2003). In addition, other significant biomarkers were either annotated based on accurate mass searches of various databases or using tandem MS spectral fragmentation patterns.

**FIGURE 7:**
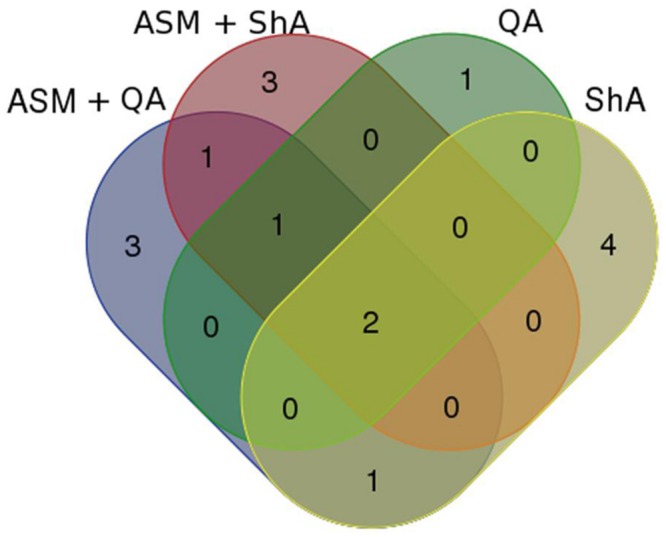
**Venn diagram showing comparison of signatory biomarkers positively associated with the various precursor/ precursor combination treatments of *C. asiatica* cells.** The biomarkers are represented by their Rt_*m/z* values as per **Tables 1–3**.

A total of 16 metabolites (13 putatively annotated) including CGA-derivatives, hydroxycinnamic acid (HCA) derivatives, terpenoids and phenolics were found to be positively correlated with the precursor/ precursor combination treatments (**Table 2**). As this is the first study to report on the use of precursor feeding in *C. asiatica* cells, a Venn diagram-based comparison was carried out in order to investigate which biosynthetic pathways are affected by each of the treatments based on the up-regulated metabolites (**Figure 7**).

**Table 2:** Annotation of significant biomarkers in precursor (QA and ShA) feeding study of *C. asiatica* cells using tandem MS and mass databases.

Rt (min)	[M-H]^-^(*m/z*)	Product ions (m/z)	Putative identification (Adduct)	Metabolite category	Database/ Tandem MS	ASM & QA	ASM & ShA	QA	ShA
4.10	221.0986	243, 207	Rishitin	Terpenoid	Knapsack	✓	✓	✓	
4.90	243.0811	193	ρ-Coumaroylserotonin	HCA conjugate	In-house	✓	✓		
5.78	375.0660	351, 191, 133	Dimethoxytyramine	HCA conjugate	In-house				✓
6.70	179.0661	164, 146	ρ-Methoxyhydrocinnamic acid	HCA	Chemspider		✓		
6.80	335.1308	293, 275, 179, 131	Caffeoylshikimic acid	HCA conjugate	In-house/ Tandem MS			✓	
7.56^∗^	367.0994	191, 173, 134	*trans*-5-Feruloylquinic acid	HCA conjugate (CGA derivative)	Tandem MS				✓
8.14	409.1481	345	Unidentified	–	-		✓		
8.22	380.9715	193, 134	Ferulic acid derivative	HCA	Tandem MS		✓		
8.38	790.5856	600, 564, 341, 249, 178	Delphinidin 3-*O*-(6 caffeoyl-beta-D-glucoside)	Anthocyanidine/HCA	In-house	✓	✓	✓	✓
9.10	311.0880	267, 191, 134	Unidentified	–	–	✓			
9.20	838.6009	609, 206, 252	Unidentified	–	–	✓			
9.28	341.0981	297	Scutellarin tetramethyl ether	Flavone	Chemspider	✓			
9.40	311.0877	267, 178	Unidentified	–	–				✓
10.46^∗^	529.1414	367, 353, 191, 179, 135	3-Caffeoyl, 5-feruloylquinic acid	HCA conjugate (CGA derivative)	Tandem MS	✓			✓
11.40	341.0996	326, 267	ρ-Coumaroyltyramine	HCA conjugate	In-house				✓
12.87	361.1587	221, 207, 192	Rishitin derivative	Terpenoid	In-house	✓	✓	✓	✓

*^∗^Biomarkers indicated with an asterix correspond to those indicated in **Table 1**.*

Evaluation of the Venn diagram revealed that the HCA-derivatives and terpenoids were up-regulated by all the elicitor/ precursor combination treatments, although the former group of metabolites appear to be well represented among the different treatments. Focusing on the CGA-derivatives, only *trans*-5-FQA and 3-C, 5-FQA were upregulated by some treatments (i.e., [ASM + QA], [ASM + ShA] and ShA, respectively) (**Table 3**).

**Table 3:** Signatory biomarkers positively associated and shared between the various elicitor and precursor combination treatments of *C. asiatica* cells (As per **Figure 7**, the Venn diagram).

Elicitor and precursor combinations	Biomarker ions (Rt_*m/z*)	Putative identification
[ASM + QA], [ASM + ShA] QA, ShA	12.87_361	Rishitin derivative
	8.38_790	Delphinidin 3-*O*-(6-caffeoyl-beta-D-glucoside)
	6.70_179	ρ-Methoxyhydrocinnamic acid
	8.14_380	Ferulic acid derivative
QA	6.80_335	Caffeoylshikimic acid
ShA	11.40_341	ρ-Coumaroyltyramine
	5.78_375	Dimethoxytyramine
	9.40_311	Unidentified
	7.56_367^∗^	Trans-5-Feruloylquinic acid

*^∗^Biomarkers indicated with an asterix correspond to those indicated in **Table 1**.*

However, the peak intensities of identified biomarker metabolites in extracts from the different treated cell samples appeared to be at varying levels (reflecting different concentrations).

### Relative Concentration of CGA Biomarkers

**FIGURE 8:**
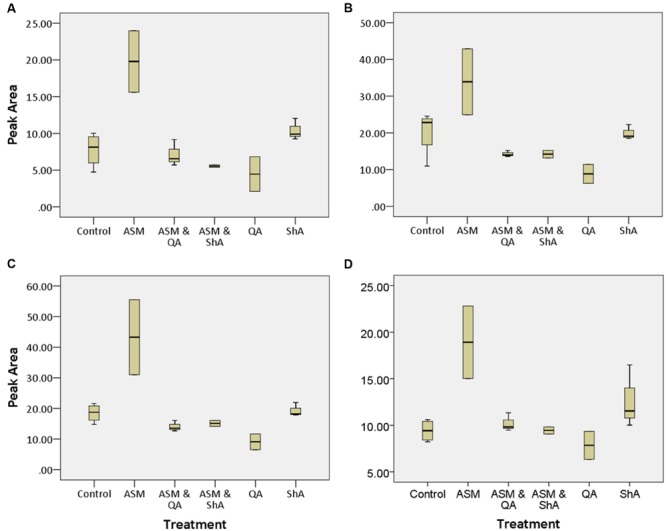
**Box and whisker plots of the relative peak areas of annotated CGA biomarkers.** Shown are the relative concentrations of **(A)** *trans*-5-FQA, **(B)** 3,5 di-caffeoylquinic acid, **(C)** 3,5-*O*-dicaffeoyl-4-*O*-malonylquinic acid (irbic acid) and **(D)** 3-caffeoyl, 5-feruloylquinic acid of *m/z* 367, 515, 601, and 529 respectively. **(A–D)** Correspond to biomarkers 1, 2, 3, and 4 respectively as shown in **Table 1**. The box and whiskers plots were constructed using the peak areas of extracted ion chromatograms (XIC) generated from the corresponding datasets.

As per relative peak area (**Figure 8**) of the annotated CGA biomarkers (**Table 1**), the treatment of *C*. *asiatica* cells with ASM resulted in approximately a twofold (100%) increase in relative concentrations of biomarkers 1–4. Interestingly, ShA-treatment also induced an increase in the relative concentration of some CGA biomarkers (1 and 4) albeit to an insignificant fold change. This could then explain the close relationship of the ASM- and ShA- treated samples on the PCA scores plot (**Figure 6A**) – and HCA plot (**Figure 6B**). The close relationship of ASM- and ShA- treated cells on the PCA plots (**Figure 6**) could be explained by that these compounds seemingly affect only CGA-derivatives in *C*. *asiatica* cells (as seen on **Table 1**). On the contrary, the [ASM + QA], [ASM + ShA] and QA treatment also resulted in the perturbation of non-CGAs (as seen in **Table 2**) and thus the samples seemingly possess a different metabolome. This would then explain the clustering of these samples further away from the ASM- and ShA- treated samples on the PCA – and HCA plots (**Figure 6B**).

#### Effect of ASM on CGAs

In general, ASM appears to have a positive effect on the annotated CGA biomarkers (**Table 1**). For some of the CGA-derivatives, the combined treatments (pre-induction followed by precursor feeding) appear to decrease their relative concentrations (**Figure 8A**). However, all the precursor/ inducer treatments appear to not induce the biosynthesis of new CGA-derivatives. According to earlier reports, the effects of exogenous application of ASM may vary depending on different pathosystems (i.e., particular host–pathogen interactions) and concentration of ASM applied to the plants (Oostendorp et al., 2001; Hong et al., 2011; Walters et al., 2013).

Based on our previous findings (Ncube et al., 2016), it is evident that the phenylpropanoid pathway in *C. asiatica* is more responsive to ASM than to SA. However, these inducers seemingly have different effects on the concentration of CGA-derivatives in *C. asiatica* cells. Therefore, the enhancement of the already existing phenylpropanoid pathway (where SA failed) is in agreement with the notion that ASM is a more effective inducer of the pathway compared to SA (Du et al., 2012; Sillero et al., 2012). In addition, the presence of only these biomarkers [1–4] in SA- and ASM-treated *C. asiatica* cells could be an indication that the enzymatic machinery of this plant predominately catalyze the biosynthesis of only these CGA-derivatives. Similarly to previous reports, *C. asiatica* cells appear to constitutively biosynthesize a relatively large pool of 3,5 diCQA [2] (Satake et al., 2007) that may be the substrate for conversion to 3,5-*O*-dicaffeoyl-4-*O*-malonylquinic acid [3] (irbic acid). Irbic acid is a species-specific compound (Antognoni et al., 2011). It is a known phenomenon that some plant species only contain a fraction of possible CGA isomers. Case in point, *Mormodica* species have been reported to synthesize only the 4-acyl CGAs (Madala et al., 2014b). On the other hand, some plants such as tobacco (Ncube et al., 2014) and coffee (Mahesh et al., 2007; Mehari et al., 2016) have been reported to biosynthesize diverse CGA isomers.

Another interesting point to note is that of all the phenylpropanoid molecules, only CGA-derivatives were found as significant biomarkers in both SA and ASM studies. This confirms that *C. asiatica* cells can be stimulated to increase the existing pool of not only centelloids but CGA-derivatives as well (Antognoni et al., 2011; Long et al., 2012; Gray et al., 2014; Maulidiani et al., 2014).

#### Effect of Precursor Feeding on CGAs

Relevant factors to consider in precursor feeding studies is the timing and the concentration of the precursor molecule to be added to a cell culture, as well as whether the particular precursor added would feed into a certain or a particular network of pathways (Jackson and Attalla, 2010; Murthy et al., 2014). Increased metabolic pools of QA and ShA might lead to corresponding increases in CGA concentrations. However, if the flux through the pathway is under strong metabolic control, negative feed-back inhibition of enzymes such as hydroxycinnamoyl-CoA shikimate/quinate hydroxycinnamoyl transferase (HCT) and hydroxycinnamoyl-CoA quinate hydroxycinnamoyl transferase (HQT) involved in CGA biosynthesis (Hoffmann et al., 2003; Niggeweg et al., 2004; Li et al., 2016), could have resulted. This could, therefore, explain the down-regulation of some of the biomarkers upon treatment with some inducer and precursor combinations (**Figure 8**). Further optimisation of the concentration of the inducer and the incubation period may result in increased biosynthesis of new CGA-derivatives. However, the treatment of the cells with just the ShA as a precursor or in combination with ASM can also increase the concentration of *trans*-5-FQA [1] and 3-C, 5-FQA [4] as shown in **Figure 8**.

This current study presents a biotechnological attempt to increase CGA accumulation (using chemical elicitation with ASM and precursors), but work with a similar objective has been carried out elsewhere using recombinant DNA approaches. Previous work of the latter resulting from the overexpression of the *HQT* gene in tomato only increased CGAs by 85% (1.85 fold) (Niggeweg et al., 2004). Constitutive expression of the artichoke *HQT* gene in tobacco resulted in a 3 fold increase in CGAs (Sonnante et al., 2010) and constitutive expression of the *AtMYB12* transcription factor gene from *A. thaliana* in potato increased CGA concentrations by 3.35-fold on average (Li et al., 2016). Thus, by either recombinant DNA manipulation or chemical elicitation, the metabolic control over CGA accumulation seems to be very tightly controlled, limiting the accumulation of CGAs.

#### General Effect of Precursor Molecules on *C. asiatica* Cells

Evidently, the exogenous application of precursors resulted in a metabolic perturbation of *C. asiatica* cells as the treatments resulted in the up-regulation of several biomarkers (**Table 2**). Furthermore, the finding of rishitin in precursor-treated cells is also of particular interest as this sesquiterpene is reportedly exclusive to the plants in the *Solanaceae* family (Tugizimana et al., 2012). In our previous report (James et al., 2013), we reported the up-regulation of rishitin in MeJA-treated *C. asiatica* cells. Therefore, this finding further verifies the role of this sesquiterpene as a phytoalexin in stressed *C. asiatica* cells. Although the aim of this study was to increase the biosynthesis of CGAs, it is interesting to note the positive effect of all the treatments on the HCA category in particular (**Table 2**). These findings further clarify the role of ASM as a highly potent elicitor of phenylpropanoids as well as the prospective use of QA and ShA in precursor feeding approaches to increase the biosynthesis of phenylpropanoids.

Moreover, these results also indicate that ASM has an effect on the terpenoid pathway of the plant as rishitin along with its derivative were found to be up-regulated by this treatment. Interestingly, in our previous work, the exogenous application of MeJA to *C. asiatica* cells resulted in the biosynthesis of both terpenoids and phenylpropanoids (Tugizimana et al., 2015). Similarly to this cross-talk effect of MeJA in *C. asiatica*, the various treatments applied here resulted in the up-regulation of both terpenoids and phenylpropanoids as well.

The co-occurrence of sesquiterpenoids, pentacyclic triterpenoids, hydroxycinnamic acid derivatives and chlorogenic acids as bio-active metabolites, thus allow for this plant to cover a wider pharmacophore space.

## Conclusion

The plant metabolomics approach, based on an UHPLC-qTOF-MS ISCID platform, was effective to investigate the effect of exogenously applied inducer of the phenylpropanoid pathway to *C. asiatica* cells along with precursor molecules of CGAs, i.e., QA and ShA. Here, a total of four CGA-derivatives were statistically found to be time- and concentration dependent signatory biomarkers associated with the ASM treatment. These biomarkers were annotated as *trans*-3-feruloylquinic acid, 3,5 di-caffeoylquinic acid, 3,5-*O*-dicaffeoyl-4-*O*-malonylquinic acid (irbic acid) and 3-caffeoyl, 5-feruloylquinic acid. However, an attempt to substantially increase the yield of these CGA-derivatives with a precursor feeding approach was not successful. Nonetheless, this approach resulted in the up-regulation of 16 metabolites including CGA- derivatives, hydroxycinnamates, phenolics and terpenoids. In comparison to our previous study, these results confirm ASM as a more potent inducer of the phenylpropanoid pathway than SA. In general, the treatment of *C. asiatica* with ASM and precursor molecules shows the potential to enhance the production of CGAs through this biotechnological approach. However, further optimization of treatment conditions could possibly result in a greater increase in CGA metabolite pools and thus a more pronounced effect in this bio-medical context.

## Author Contributions

Conceived and designed the research: ID. Performed the experiments: EN and PS. Analyzed the data: EN, NM, and ID. Interpreted the data: ID. Wrote the paper: EN, NM, and ID.

## Conflict of Interest Statement

The authors declare that the research was conducted in the absence of any commercial or financial relationships that could be construed as a potential conflict of interest.

## Footnotes

1 https://xcmsonline.scripps.edu

2 www.ibm.com/SPSS\_Statistics

3 www.chemspider.com

4 http://dnp.chemnetbase.com/

5 http://kanaya.naist.jp/KNApSAcK/

6 www.taverna.org.uk

## Supplementary Material

FIGURE S1 **UHPLC-MS analysis of *C. asiatica* cells extracts corresponding to the inducer (ASM) and precursor (QA and ShA) combinations as indicated in A–D**.

FIGURE S2 **Multivariate data models (OPLS-DA scores and OPLS-DA S-plots indicated by 1 and 2 respectively), representative of metabolite changes occurring in *C. asiatica* cells corresponding to the inducer and precursor combinations as indicated: **(A)** [ASM and QA] vs. Control, **(B)** [ASM and ShA] vs. Control, **(C)** QA vs. Control, and **(D)** ShA vs. Control**.

FIGURE S3 **UHPLC-QTOF-MS fragmentation spectra of CGAs: **(A)***trans*-3-feruloylquinic acid, **(B)** 3,5 di-caffeoylquinic acid, **(C)** 3,5-*O*-di-caffeoyl-4-*O*-malonylquinic acid (irbic acid), and **(D)** 3-caffeoyl, 5-feruloylquinic acid**.
