# Supplementary figures and images for: Stimulatory Effects of Acibenzolar-S-Methyl on Chlorogenic Acids Biosynthesis in Centella asiatica Cells
Source: Front Plant Sci. 2016 Sep 28;7:1469. doi: 10.3389/fpls.2016.01469 (PMC5040108; doi:10.3389/fpls.2016.01469)

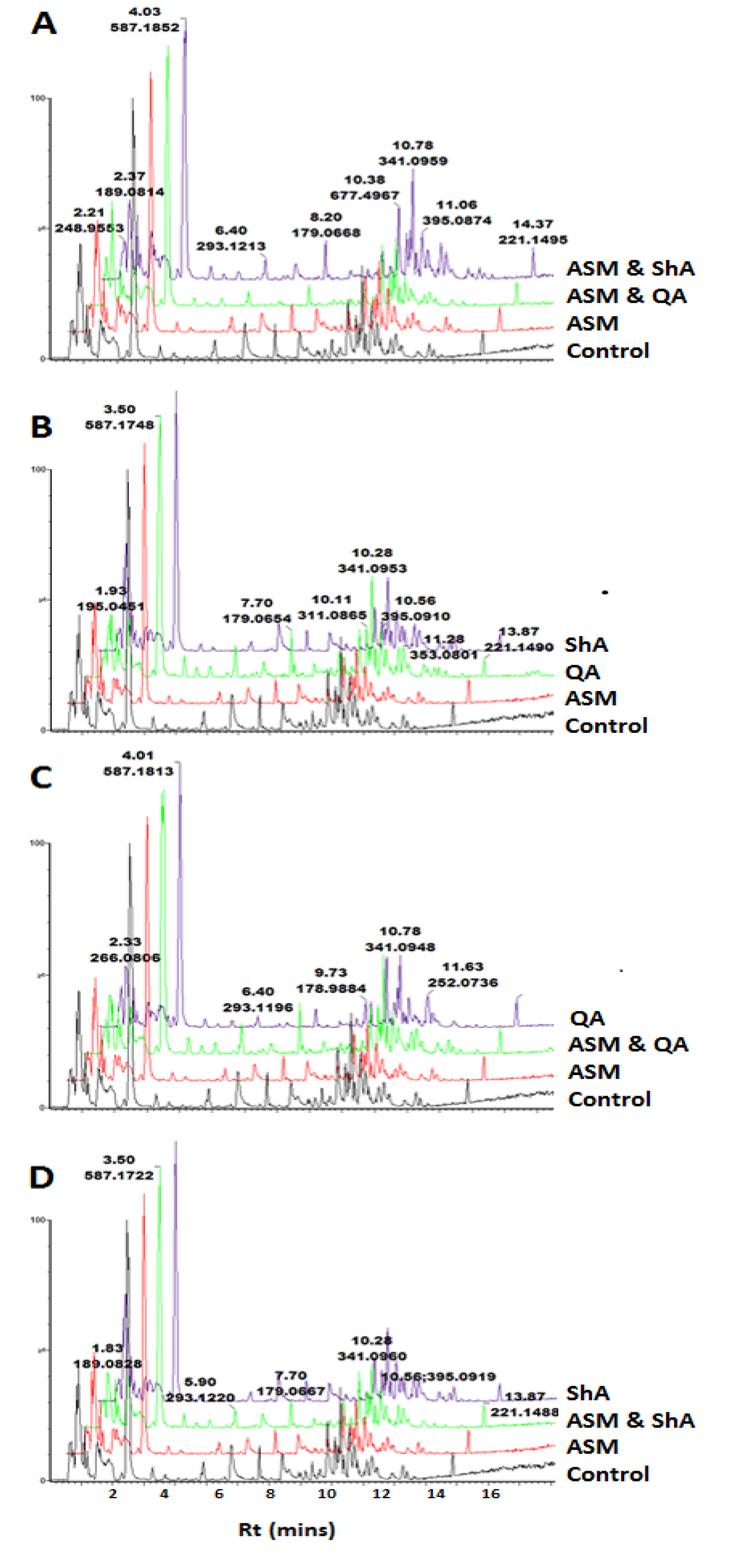

Supplement: FIGURE S1 — UHPLC-MS analysis of C. asiatica cells extracts corresponding to the inducer (ASM) and precursor (QA and ShA) combinations as indicated in A–D. [file Image_1.TIF]

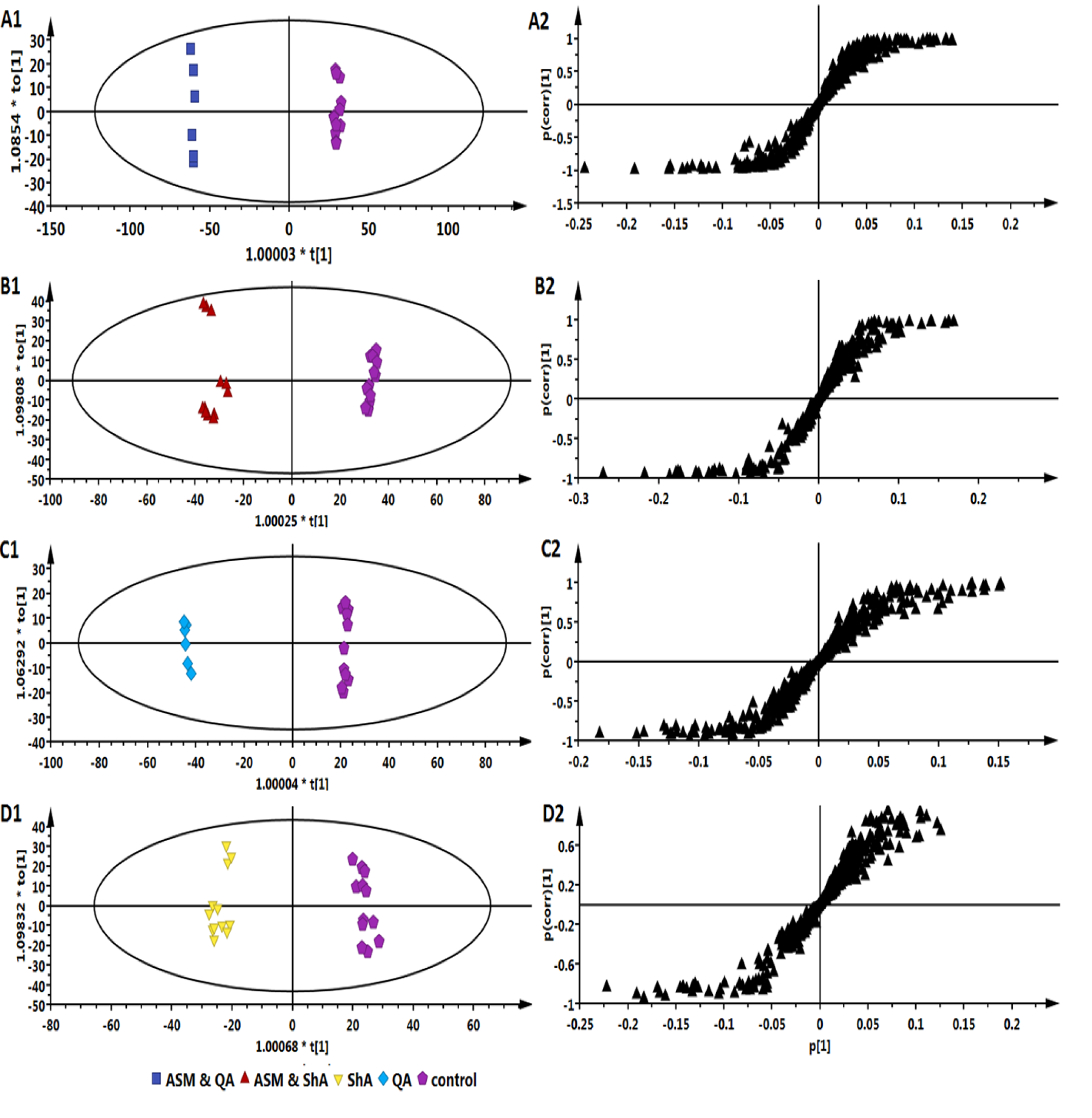

Supplement: FIGURE S2 — Multivariate data models (OPLS-DA scores and OPLS-DA S-plots indicated by 1 and 2 respectively), representative of metabolite changes occurring in C. asiatica cells corresponding to the inducer and precursor combinations as indicated: (A) [ASM and QA] vs. Control, (B) [ASM and ShA] vs. Control, (C) QA vs. Control, and (D) ShA vs. Control. [file Image_2.TIF]

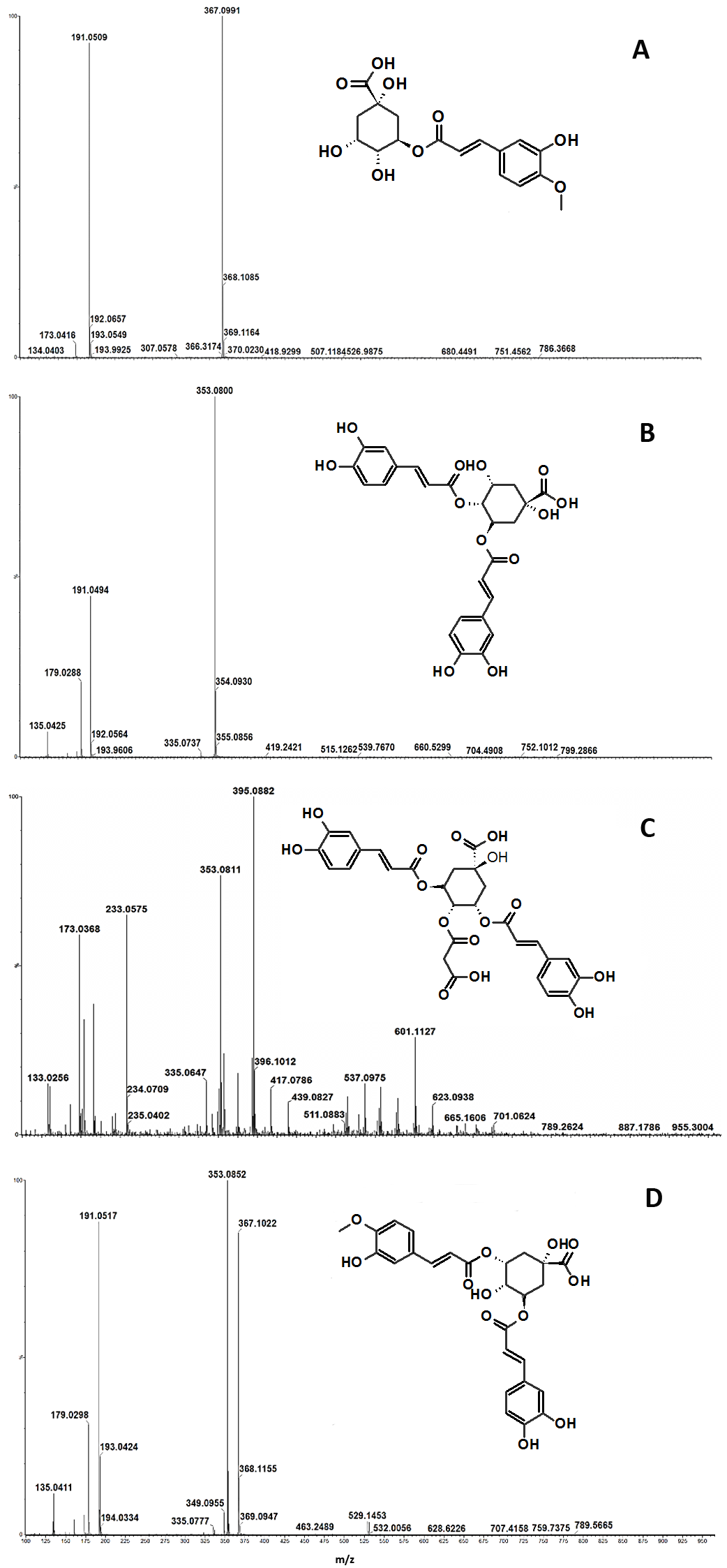

Supplement: FIGURE S3 — UHPLC-QTOF-MS fragmentation spectra of CGAs: (A)trans-3-feruloylquinic acid, (B) 3,5 di-caffeoylquinic acid, (C) 3,5-O-di-caffeoyl-4-O-malonylquinic acid (irbic acid), and (D) 3-caffeoyl, 5-feruloylquinic acid. [file Image_3.TIF]
