# Supplementary material for: Stimulatory Effects of Acibenzolar-S-Methyl on Chlorogenic Acids Biosynthesis in Centella asiatica Cells
Source: Front Plant Sci. 2016 Sep 28;7:1469. doi: 10.3389/fpls.2016.01469 (PMC5040108; doi:10.3389/fpls.2016.01469)
Supplement: Supplementary file 4 [file Table_1.PDF]

Table S1: R^2X (cum) and Q^2 (cum) values for each generated PCA model of ASM time – and concentration study.

	R^2X cum (%)	Q^2 cum (%)
**Figure 3A_ASM time study**	55	30
**Figure 4A_ASM concentration study**	59	45
**Figure 6_Precursor feeding study**	59	47

Table S2: CV Anova p-values for each generated OPLS-DA model of the time – and concentration study, as well as the precursor feeding studies.

	OPLS-DA	CV Anova *p*-value
**ASM**	Figure 3_Time study	> 0.001
	Figure 4_Concentration	> 0.001
**Precursor feeding**	Figure S2A_[ASM + QA]	> 0.001
	Figure S2B_[ASM + ShA]	> 0.0001
	Figure S2C_QA	> 0.0001
	Figure S2D_ShA	> 0.0001
